# Venous Excess Ultrasound for Fluid Assessment in Complex Cardiac Patients With Acute Kidney Injury

**DOI:** 10.7759/cureus.66003

**Published:** 2024-08-02

**Authors:** Shubhangi Kanitkar, Kritika Soni, Bhumika Vaishnav

**Affiliations:** 1 Internal Medicine, Dr. D. Y. Patil Medical College Hospital and Research Centre, Pune, IND

**Keywords:** point-of-care-ultrasound, : acute kidney injury, venous excess ultrasound score, vexus, pocus

## Abstract

Introduction: The introduction of point-of-care ultrasound (POCUS) into clinical practice has revolutionized bedside hemodynamic assessment in recent years. POCUS has expanded its utility to include evaluating and grading venous congestion through Doppler analysis of venous blood flow. This innovative technique, VExUS (venous excess ultrasound), comprehensively evaluates venous congestion across multiple sites, including the inferior vena cava (IVC), hepatic vein, portal vein, and intrarenal vasculature. The aim of the current study was to determine whether venous excess ultrasound can help guide fluid therapy in complex patients with acute kidney injury (AKI) in addition to the standard physical examination and imaging.

Methods: Our current study shows instructive 18 clinical adult cases (enrolled between January 2024 and May 2024) to determine whether venous excess ultrasound can help guide fluid therapy in complex cardiac patients with acute kidney injury, in addition to the standard physical examination and imaging.

Results: VExUS was pivotal in guiding fluid therapy in all complex patients with AKI and suspected right ventricular dysfunction. By integrating VExUS findings with clinical data and cardiac ultrasound results, clinicians were able to make patient-favouring decisions regarding fluid management, diuresis, and vasopressor therapy, addressing critical aspects of conditions such as septic shock, heart failure, and acute kidney injury.

Conclusions: In our study of VExUS in sick patients with AKI, we concluded that VExUS proved to be a valuable tool for fluid assessment and management. By providing real-time visualization of venous congestion, VExUS allowed for more precise and individualized fluid management strategies. This led to improved decision-making regarding fluid administration and removal, helping to prevent both fluid overload and hypovolemia. Consequently, the use of VExUS contributed to better clinical outcomes in patients with AKI, demonstrating its potential as a critical component in the management of fluid balance in this vulnerable patient population.

## Introduction

In critically ill patients, monitoring hemodynamic parameters is crucial for optimizing perfusion and preventing organ dysfunction [1,2]. Traditionally, the focus has been on maintaining adequate cardiac output and arterial blood pressure. However, the significance of venous congestion, often overlooked, plays a significant role in organ perfusion [3,4]. Traditional hemodynamic methods like central venous pressure (CVP) may not capture the complexity of venous congestion, emphasizing the need for advanced techniques like point-of-care ultrasound (POCUS) and innovative tools like venous excess ultrasound (VExUS) grading. Regular monitoring and tailored interventions based on VExUS grading can significantly avoid indiscriminate fluid loading and preserve optimal perfusion dynamics, enhancing patient outcomes and reducing mortality risks.

In recent years, the paradigm of assessing venous congestion has significantly transformed with the advent of VExUS. This innovative bedside, four-part ultrasound-based approach, pioneered by Beaubien-Souligny et al., presented a revolutionary method for evaluating venous blood flow through key vasculatures, including the inferior vena cava (IVC), hepatic vein, portal vein, and intrarenal vasculature [5]. The culmination of these measurements results in a VExUS grade, a novel indicator for venous congestion. This approach originated from a meticulous post-hoc analysis correlating ultrasound grading parameters with the risk of acute kidney injury (AKI) development in cardiac surgery patients [5-7]. The VExUS score is a clinical tool with a four-part ultrasound approach to assess venous congestion by evaluating IVC diameter, hepatic vein Doppler, portal vein Doppler, and intrarenal vein Doppler. Venous Doppler imaging assesses the return flow pattern of venous congestion once it is identified. Venous congestion can be evaluated at the liver, the portal, and the intra-renal vein. Venous flow in the hepatic vein provides information on the mechanics of the right heart-filling patterns. Flow patterns on the portal and intra-renal veins offer information on the degree of pressure transfer to the peripheral organs [5,6]. 

POCUS combined with the VExUS score offers invaluable advantages in assessing and managing venous congestion across various medical conditions. One of its primary benefits lies in early detection, enabling clinicians to identify venous congestion promptly in patients with heart failure, liver disease with kidney disease, and fluid overload. This early detection is pivotal as it facilitates timely interventions, potentially averting complications. Moreover, POCUS with VExUS is non-invasive, ensuring patient safety by avoiding exposure to ionizing radiation or contrast agents. This makes it a suitable option for repeated assessments of critically ill patients. VExUS provides real-time images, allowing clinicians to monitor changes in venous congestion over time. This is especially important in dynamic clinical situations where a patient's condition may change rapidly. VExUS is conducted at the patient's bedside and provides immediate insights, aiding clinicians in making swift and informed decisions, a vital aspect in critical care settings [5-7]. Additionally, the VExUS score assists in stratifying patients based on congestion severity, enabling tailored treatments and interventions. VExUS may play a crucial role in preventing unnecessary interventions by distinguishing between venous congestion and other conditions with similar symptoms. Once venous congestion is identified, effective treatment strategies, such as diuretic therapy or fluid management adjustments, can be employed. The VExUS protocol's focus on assessing the venous-to-arterial blood flow ratio in the renal and hepatic veins differentiates it. Additionally, there are reports of IVC diameters and collapsibility indexes with significant correlations with CVP [5-8]. The combination of VExus with IVC diameters and collapsibility indices with CVP correlations allows clinicians to diagnose the underlying cause of congestion, whether cardiogenic factors like heart failure or non-cardiogenic issues like renal dysfunction. In short, POCUS with the VExUS score is an indispensable tool that enhances the precision of diagnosis, guides treatment, and ultimately improves patient outcomes in the complex realm of venous congestion management [5-9]. 

## Materials and methods

Aim

The study aims to determine whether venous excess ultrasound can help guide fluid therapy in complex patients with acute kidney injury, in addition to the standard physical examination and imaging, and whether it can be of educational value for further prospective studies.

Study type and duration

A case-series study was conducted in a tertiary hospital from January 2024 to May 2024.

Inclusion and exclusion criteria

Individuals diagnosed with AKI as per the KDIGO classification and complex clinical conditions, such as suspected right ventricular pathology and dilated IVC with challenging fluid management scenarios as per the treating physician, were enrolled in the study. Participants aged 18 years and older were included, provided they gave informed consent.

Exclusion criteria encompassed non-consenting patients, individuals below 18 years, and those with known allergies to ultrasound gel or related substances. Additionally, patients in critical condition, those with inadequate imaging windows, pregnant or breastfeeding women, individuals with chronic atrial fibrillation, mechanical cardiac assistance, uncontrolled low blood pressure, or undergoing specific medical interventions like veno-venous hemofiltration or dialysis were excluded from the study. These criteria were established to ensure the study's scientific integrity, ethical compliance, and participant safety.

Data collection

The baseline patient information recorded includes patient demographics, existing co-morbidities, and the reason for admission. Clinical assessments, including hemodynamic factors, fluid balance, and ventilator parameters, including the type of ventilation (invasive or non-invasive), FiO_2_ (fraction of inspired oxygen), tidal volume, respiratory rate, and end-expiratory pressure, were analyzed. Biological parameters vital for monitoring the patient's physiological state encompassed haemoglobin levels, hematocrit levels, sodium levels, arterial lactate levels, and daily serum creatinine levels. Approval from the Institutional Review Board was obtained for the current study.

Ultrasound measurements

**Figure 1:**
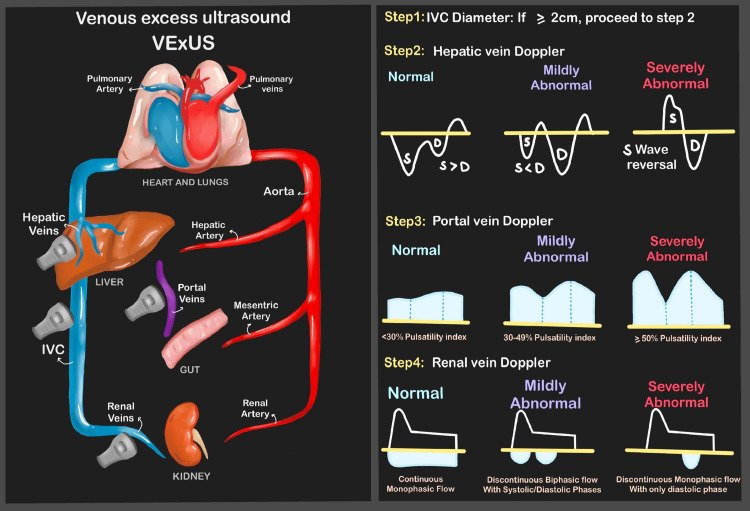
VExUS grading Interpretation of VExUS Grading: Grade 0: IVC <2 cm = NO congestion Grade 1: IVC >2 cm with any combo of normal or mildly abnormal patterns = MILD congestion Grade 2: IVC >2 cm and ONE severely abnormal Pattern = MODERATE congestion Grade 3: IVC >2 cm and >2 severely abnormal patterns = SEVERE congestion

The assessment of the VExUS score and meticulous ultrasound measurements were conducted in adherence to established protocols. VExUS grading and Doppler patterns are shown in Figure 1.

Recent studies on the VExUS application are summarized in Table 1.

**Table 1:** A table depicting recent studies on VExUS application

Study type	n	Conclusion	Authors
1. Study type: Post-hoc analysis of a single-centre prospective study. Aims: Assessing systemic congestion through point-of-care ultrasound: the evolution of the venous excess ultrasound grading system.	145	Severe congestion, defined as a dilated IVC (Grade 3 in the VExUS system), is strongly associated with subsequent AKI (hazard ratio: 3.69, confidence interval 1.65–8.24, p = 0.001; positive likelihood ratio: 6.37, confidence interval 2.19–18.50).	Beaubien-Souligny et al. [5]
2. Study type: A prospective cohort Study. Aims: The integration of Inferior Vena Cava Diameter, Hepatic Venous Flow, and Portal Vein Pulsatility Index into Venous Excess Ultrasound Score (VExUS Score) demonstrates promising potential for predicting acute kidney injury in patients afflicted with cardiorenal syndrome.	30	The resolution of acute kidney injury showed a correlation with the improvement in VExUS grade (p-value = 0.003). A noteworthy relationship was observed between changes in VExUS grade and fluid balance (p-value = 0.006).	Bhardwaj et al. [6]
3. Study type: A cross-sectional pilot study. Aims: The practicality and effectiveness of employing the Venous Excess Ultrasound Score for identifying and classifying elevated central venous pressure in critically ill pediatric patients.	33	VExUS score severity was strongly associated with CVP elevation p < 0.001 in critically ill children. Higher VExUS scores (>1) correlated with poorer outcomes (mortality, ICU admission) compared to lower score (p = 0.0015).	Menendez-Suso et al. [7]
4. Study type: Observational study. Aims: VExUS score performed on ED septic patients prior to receiving fluids with chart review done to determine if there is an association with poorer outcomes.	150	Composite outcome (mortality, ICU admission, or rapid response activation): VExUS score of 0: 31.6% of patients VExUS score of 1: 47.6% of patients VExUS score >1: 67.7% of patients (p = 0.0015).	Rolston et al. [8]
5. Study type: Prospective observational study. Aims: Evaluation of predictive scores (portal pulsatility index, renal venous impedance index, VExUS) for fluid depletion caused by diuretics.	81	The Portal Pulsatility Index and Renal Venous Impedance Index demonstrated better predictive capabilities as compared to VExUS. The baseline VExUS score exhibited limited predictability for diuretic-induced fluid depletion (AUC of 0.66, 95% CI 0.53–0.79, p = 0.012) p = 0.012)	Guinot et al. [9]

The VExUS score for AKI patients was defined as congestive (VExUS ≥ 2): patients with a VExUS score of 2 or higher, indicating moderate to severe renal congestion; or non-congestive (VExUS < 2): patients with a VExUS score below 2, indicating no congestion.

Ultrasound examinations were performed in conjunction with simultaneous ECG monitoring to precisely identify the phases of the cardiac cycle. The IVC diameter was precisely measured at 1.0 cm from its junction with the right atrium in the subcostal view, with both maximum and minimum diameters recorded. The percentage change in diameter was calculated, offering valuable data for assessment. Additionally, pulsed-wave (PW) Doppler assessment in the liver hilum measured the maximum velocity (*V*_max_) and minimum velocity (*V*_min_) of the portal vein [10]. These values were crucial for computing the Portal Pulsatility Index (PI) using the formula PI = (*V*_max_ − *V*_min_)/*V*_max_, providing essential insights into hemodynamic status. PW Doppler measurements were also conducted in the interlobar veins of each kidney's upper, median, and lower segments [11]. The resulting intrarenal venous flow patterns were meticulously classified according to established waveform patterns, enhancing the depth of the analysis [11,12]. The ultrasound findings guided fluid therapy, diuretic administration, and other interventions to optimize the patient's fluid balance.

Definition and outcomes

The VExUS score was determined at four crucial junctures: within 24 hours of admission, after the first day (between 24 and 48 hours), following the second day (between 48 and 72 hours), and at the point of discharge [13]. Patients were categorized into congestive (VExUS ≥ 2) or non-congestive (VExUS < 2) groups, with grade 2 indicating moderate congestion and grade 3 marking severe congestion.

AKI stages were defined as follows: Stage 1 was defined as an increase in serum creatinine (SCr) by ≥ 0.3 mg/dL or to ≥ 1.5 times baseline or reduced urine output (< 0.5 mL/kg/h) for 6-12 hours. Stage 2 was defined as a serum creatinine increase to ≥ 2.0-2.9 times baseline or reduced urine output for ≥ 12 hours. Stage 3 was defined as serum creatinine up to 3.0 times baseline, serum creatinine increased to ≥4.0 mg/dL, initiation of kidney replacement therapy, minimal urine output for ≥ 24 hours, or anuria for ≥12 hours.

Baseline serum creatinine, representing kidney function before admission, was determined within three months before hospitalization. If the baseline serum creatinine was unavailable and there was no history of renal disease, we estimated it to be 1 mg/dL. Furthermore, the study examined the 28-day mortality rate, recording the number of patients who passed away within 28 days of their admission.

## Results

A total of 18 patients (n = 3 representative cases shown below and n = 15 cases shown in Tables 2-4) were studied (median age 65; out of 15, 9 were male (60%)). In addition to the standard physical examination and diagnostic imaging, VExUS was performed on all the patients. The clinical cases and the utilization of VExUS played a crucial role in guiding the management and ultimately improving the patient's condition. We present three representative cases where VExUS-guided therapy.

**Table 2:** Below is the table of patients where VExUS assessments helped in guiding the treatment in five patients with acute kidney injury and multisystemic involvement in the form of heart failure/cirrhosis/pulmonary edema

	Clinical presentation	VExUS assessment	VExUS-guided treatment
1	A 55-year-old female with a history of systolic heart failure presented with worsening fatigue and reduced urine output	Severe congestion, grade 3; severely dilated IVC	Diuretics and ACE inhibitors initiated, guided by VExUS findings, and prevented acute kidney injury. Regular follow-ups showed stabilized renal function.
2	A 63-year-old female with a history of heart failure and chronic obstructive pulmonary disease presented with fatigue and decreased exercise tolerance	Chronic venous congestion (grade 2); dilated IVC	Optimized heart failure management and diuretic adjustment led to improved cardiac output and alleviation of symptoms.
3	A 64-year-old female with a history of diabetes and coronary artery disease was admitted with acute onset dyspnea and hypotension (indicating acute left ventricular dysfunction)	Severe congestion (grade 3); markedly dilated IVC	Prompt initiation of inotropic support and diuretics. Significant improvement in cardiac function and resolution of symptoms.
4	A 70-year-old male with a history of atrial fibrillation and liver cirrhosis presented with abdominal distension and oliguria	Congestion (grade 2); moderately dilated IVC	Paracentesis and diuretic therapy done. Reduction in ascites and improved renal perfusion and serum creatinine.
5	A 75-year-old male with a history of hypertension and end-stage renal disease on hemodialysis presented with fluid overload and worsening renal function	Severe congestion (grade 3); markedly dilated IVC	Aggressive ultra-filtration during hemodialysis sessions and initiation of appropriate cardiac medications. Improved fluid balance and renal function.

**Table 3:** VExUS, an accurate assessment of venous congestion guided the initiation of diuretic therapy in five patients with acute kidney injury and right heart strain or failure/pulmonary hypertension/COPD/multisystem involvement

	Clinical presentation	VExUS assessment	VExUS-guided treatment
1	A 65-year-old male, post-pulmonary embolism, presented with right-sided heart failure symptoms.	Severe congestion (grade 3); severely dilated IVC	Aggressive diuresis with furosemide was started. Significant reduction in IVC diameter and alleviation of respiratory distress within a week.
2	A 78-year-old female, a known case of pulmonary hypertension, developed acute kidney injury and bilateral lower limb edoema.	Severe congestion (grade 2); severely dilated IVC	Furosemide infusion was commenced. Gradual improvement in renal function and resolution of systemic congestion.
3	A 72-year-old male with COPD exacerbation developed acute-on-chronic kidney injury and bilateral leg swelling.	Severe congestion (grade 2); severely dilated IVC	Furosemide infusion was initiated. Improved oxygenation, relief of leg swelling, and gradual recovery of renal function.
4	A 60-year-old male with a complex medical history, including diabetes, hypertension, and chronic kidney disease, presented with multisystem failure.	Severe congestion (grade 2); severely dilated IVC	Diuretic therapy and cardiac optimization, helped manage the patient's complex condition and prevent further deterioration.
5	A 65-year-old male presented with acute chest pain and hypotension. Initial suspicion was acute coronary syndrome, but with VExUS evaluation, the diagnosis of acute right heart strain was made.	Severe congestion (grade 2); severely dilated inferior vena cava	Timely intervention with thrombolytic therapy saved the patient's life.

**Table 4:** VExUS assessments provided valuable insights into congestion dynamics, allowing precise adjustment of diuretic therapy and facilitated efficient decongestion, improving renal function and overall clinical status in five patients with acute kidney injury and chronic heart failure/cirrhosis/pulmonary edema/post-cardiac surgery

	Clinical presentation	VExUS assessment	VExUS-guided treatment
1	A 65-year-old female with chronic heart failure experienced worsening dyspnea and peripheral edoema	Severe congestion (grade 2) dilated IVC	Precise adjustment of diuretic therapy, guided by VExUS, alleviated congestion without inducing hypovolemia. This prevented diuretic resistance, improved renal function, and restored quality of life significantly.
2	A 50-year-old male with advanced liver cirrhosis presented with massive ascites and hepatorenal syndrome (serum creatinine 2.3 mg/dl)	Hepatic and renal congestion (grade 3) dilated hepatic veins and IVC	Diuretic therapy based on VExUS prevented further deterioration, reduced ascites, prevented further hepatic decompensation, and improved renal function, enabling the patient to be a candidate for liver transplantation.
3	A 64-year-old female diabetic patient, post-cardiac surgery, developed fluid overload and oliguria [0.5 ml/kg/hr × 12 hours]	Moderate congestion (grade 2) dilated hepatic veins	Customized diuretic adjustments guided by VExUS prevented complications like pleural effusion, ensuring efficient decongestion. The patient's renal function improved, and VExUS monitoring played a vital role in preventing recurrent fluid accumulation.
4	A 58-year-old male hypertensive patient presented with acute pulmonary edema and worsening renal function (serum creatinine 2.6 mg/dl)	Severe congestion (grade 3) dilated IVC	VExUS-guided diuretic optimization prevented diuretic resistance, rapidly resolving pulmonary edema, and improving renal function. The patient's clinical status stabilized, and VExUS assessments confirmed sustained decongestion.
5	A 70-year-old male with chronic kidney disease and recurrent hypertensive crises and acute kidney injury (serum creatinine 3.2 mg/dl)	Severe congestion (grade 2) dilated IVC	VExUS-guided diuretic therapy prevented diuretic resistance, enabling gradual but efficient decongestion. Renal function stabilized, and the overall clinical status improved.

Case series

Instructive Case and Case Series-I

**Figure 2:**
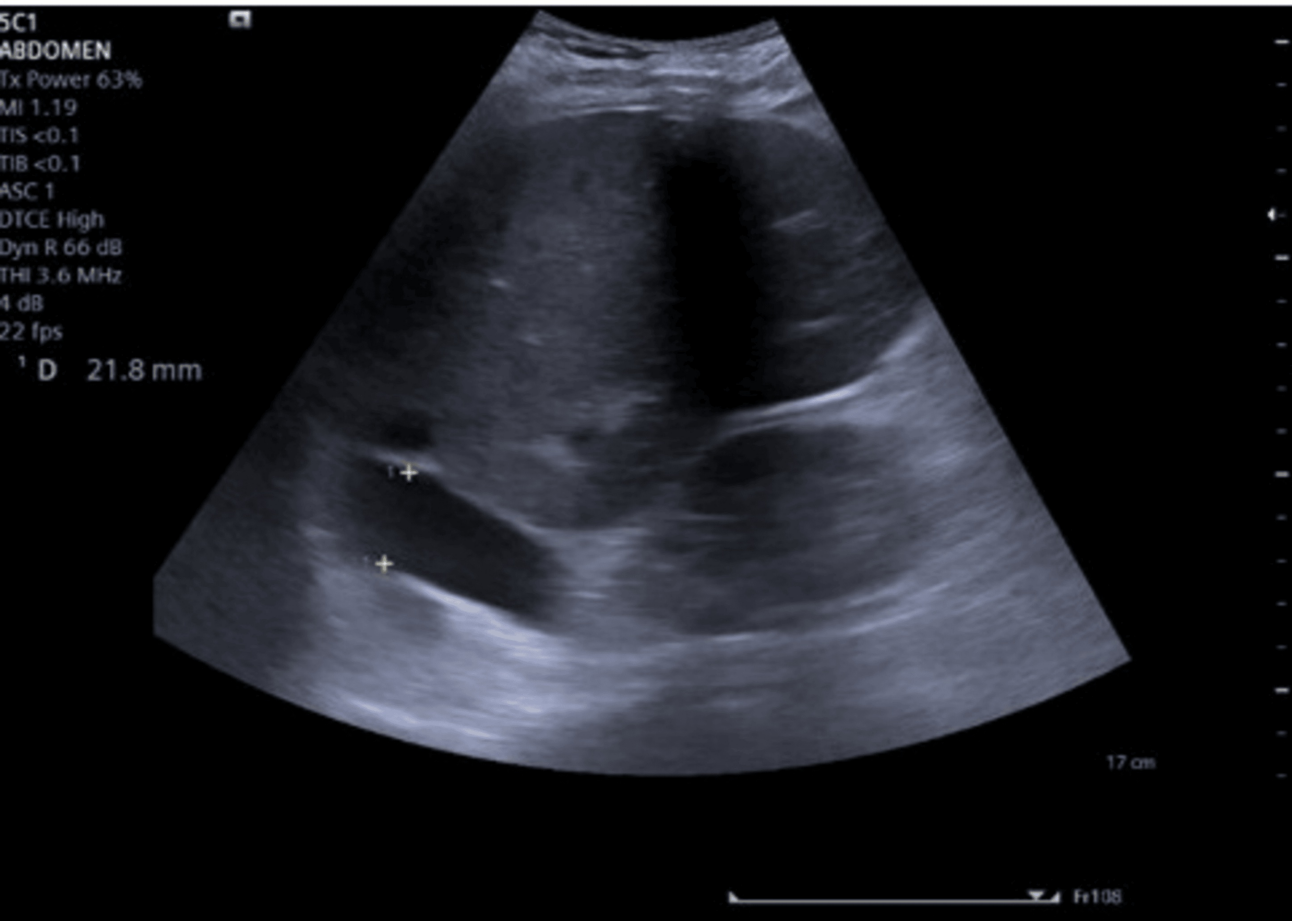
VeXUS congestion Grade 3 (severe) and inferior vena cava (IVC) diameter markedly dilated IVC (>2 cm) measuring 21.8 mm

**Figure 3:**
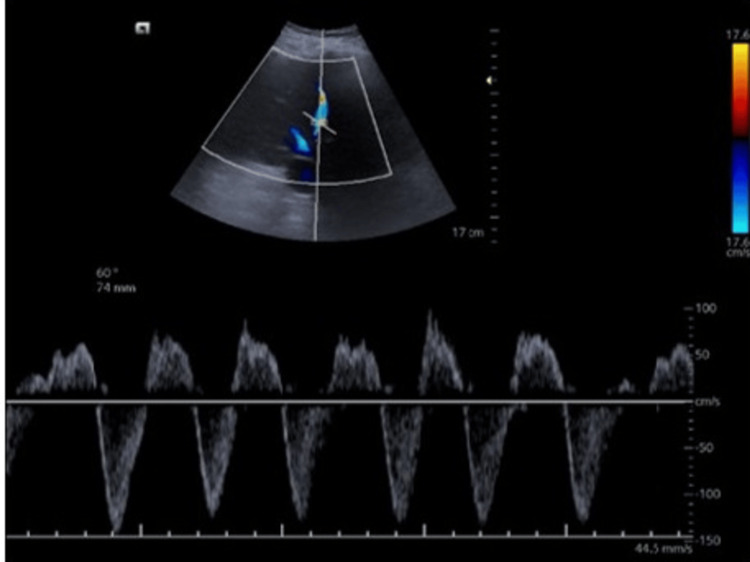
Hepatic vein Doppler showing reverse systolic phase considered severe abnormality

**Figure 4:**
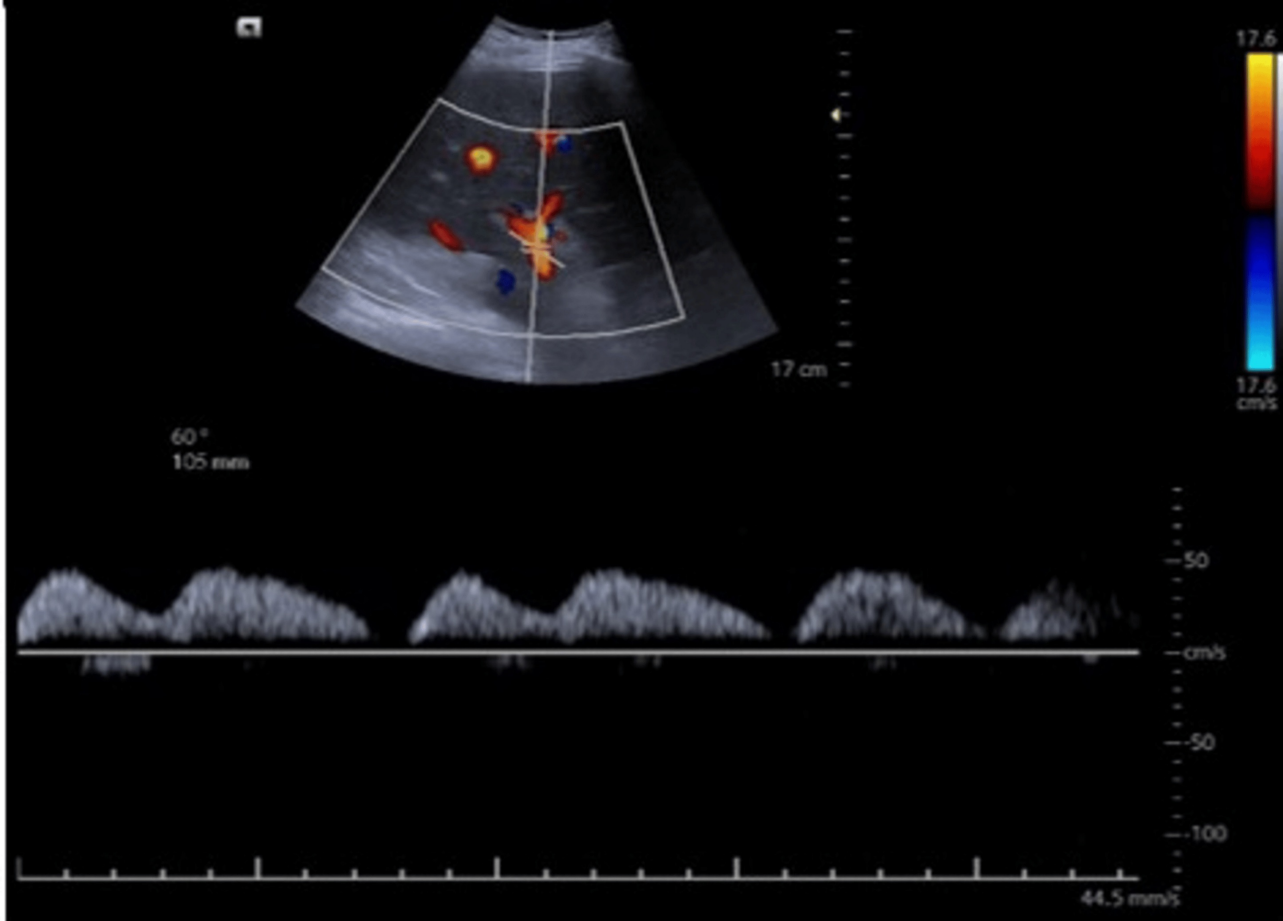
Portal vein Doppler showing diastolic flow reaching the baseline, indicative of increased pulsatility index of >0% considered severe abnormality

**Figure 5:**
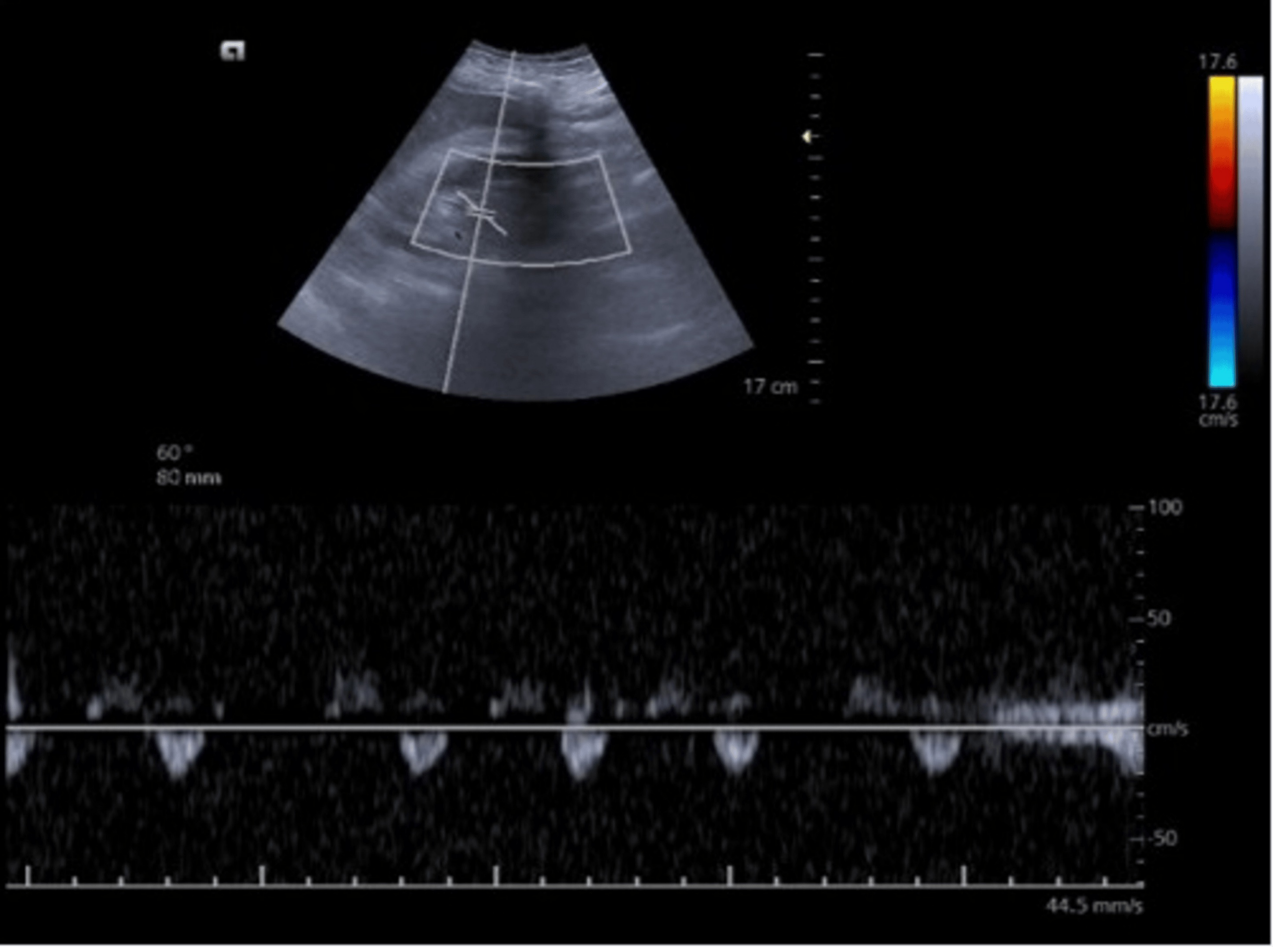
Intra-renal venous Doppler showing severe abnormality as suggested by only diastolic flow tracing below the baseline

Clinical presentation and POCUS results: A 70-year-old male with a history of alcoholic cirrhosis and severe congestive cardiac failure presented with worsening respiratory distress and acute kidney injury. On examination, the patient exhibited tense ascites and anasarca. Hemodynamic parameters indicated signs of fluid overload and compromised cardiac function. Laboratory tests revealed an elevated serum creatinine level of 2.1 mg/dl (0.7-1.1), indicating acute kidney injury. His POCUS examination through VExUS was revealed (Figures 2-5).

Treatment approach: The patient underwent daily abdominal paracentesis to alleviate ascites and received intravenous furosemide to manage fluid overload. The serum creatinine levels decreased significantly to 0.8 mg/dl over a week, indicating improved renal function. The tailored intervention guided by the VExUS assessment resulted in a positive clinical response. The patient's respiratory distress was alleviated, ascites were reduced, and renal function significantly improved. Regular monitoring was continued to ensure the stability of the patient's condition. Follow-up assessments included regular renal function tests, echocardiography, and clinical evaluations to track the patient's cardio-renal status progress and adjust the treatment plan accordingly.

Discussion: This case study investigates the effectiveness of VExUS assessment in distinguishing between chronic venous congestion and acute left ventricular dysfunction, guiding precise therapeutic interventions for patients with complex cardio-renal syndromes. Integrating such techniques into routine clinical practice is essential for improving diagnostic accuracy, tailoring treatment strategies, and ultimately enhancing patient outcomes and quality of life. Patients managed on similar lines with the help of VeXUS are shown in Table 2.

Instructive Case 2 and Case Series II

Clinical presentation and POCUS results: An 84-year-old male with significant pulmonary hypertension and mitral regurgitation was admitted with acute kidney injury and respiratory distress. Upon admission, the patient exhibited systemic congestion, including edoema and bilateral crackles in both lower lung lobes. Initial vital signs showed a blood pressure of 140/90 mm Hg. He received oxygen via nasal prongs and received epinephrine and dobutamine infusions to maintain hemodynamic stability. His serum creatinine level was elevated at 2 mg/dl (0.7-1.1), indicating acute kidney injury. VExUS evaluation revealed inferior vena cava diameter: severely dilated, fixed, and congestion grade: 3 (severe).

Treatment approach: He received oxygen via nasal prongs and received epinephrine and dobutamine infusions to maintain hemodynamic stability. Based on VExUS findings, a furosemide infusion was initiated, leading to progressive improvement in the patient's oxygen requirement, hemodynamic and acute kidney injury over the subsequent week.

Discussion: This case highlights the importance of VExUS evaluation in managing complex cases where co-existent pulmonary hypertension complicates the interpretation of elevated CVP and IVC diameters. Through VExUS, an accurate assessment of venous congestion was possible, which, in turn, guided the initiation of diuretic therapy. This approach successfully managed the patient's congestive heart failure, improving his clinical condition. Patients managed on similar lines with the help of VeXUS are shown in Table 3.

Instructive Case-3 and Case Series

Clinical presentation and POCUS results: A 60-year-old male with a history of pulmonary hypertension was admitted with worsening anasarca, dyspnea, and acute kidney injury (serum creatinine 2.3 mg/dl (0.7-1.1)). Initial intravenous furosemide provided only a partial response. Daily VExUS assessments revealed: IVC diameter: dilated; congestion grade: 3 (severe).

Treatment approach: Based on the VExUS assessment findings, aggressive diuretic therapy was initiated. The patient responded well to the diuretic treatment, and subsequent VExUS assessments showed improvement in congestion grade and reduced IVC dilation. Consequently, the patient's clinical status improved significantly, leading to a decrease in serum creatinine levels from 2.3 mg/dl to 1 mg/dl (0.7-1.1).

Discussion: Implementing VExUS assessments provided valuable insights into congestion dynamics, allowing precise adjustment of diuretic therapy, preventing diuretic resistance, facilitating efficient decongestion, and improving renal function and overall clinical status. VExUS assessments offer a novel approach for real-time monitoring of congestion levels, enabling tailored diuretic therapy. Patients managed on similar lines with the help of VeXUS are shown in Table 4.

## Discussion

VExUS is a valuable tool in managing fluid therapy for complex patients, particularly those with AKI combined with right heart failure, pulmonary hypertension, COPD, or pulmonary edema. VExUS assesses venous congestion using ultrasound to evaluate the IVC and hepatic, portal, and renal veins [1-4]. This score helps in identifying fluid overload, which is critical in patients where fluid balance is challenging. In patients with AKI and right heart failure or pulmonary hypertension, excessive fluid can worsen heart function and kidney injury. VExUS aids in tailoring fluid therapy by providing real-time data on venous congestion, thus preventing fluid overload. For COPD and pulmonary edoema patients, accurate fluid management is crucial to avoid exacerbating respiratory issues. By using VExUS, clinicians can make informed decisions on fluid administration or restriction, diuretic use, and other interventions. This individualized approach helps optimize fluid status, improve patient outcomes, and reduce the risk of complications related to both under- and over-hydration. Thus, VExUS is an essential non-invasive method for managing fluid therapy in these complex patient populations [1-6].

Often, detecting venous congestion linked to potential end-organ dysfunction necessitates invasive monitoring or ultrasound assessments. However, the practical difficulty revolves around judicious fluid administration and removal. An isolated analysis of IVC ultrasound may not give all the information needed. A detailed VExUS may give more practical information to patients with suspected systemic venous congestion. While VExUS may not offer definitive guidance on fluid necessity, it can serve as a guidepost, signalling when to halt fluid resuscitation and pinpointing patients likely to benefit from fluid removal. The traditional emphasis on cardiac output or forward flow in fluid resuscitation overlooks the pivotal role of venous congestion. Extensive literature indicates that beyond a certain point, venous congestion, as characterized by parameters such as CVP or venous Doppler indices, negates the benefits of augmented forward flow [5,10,11,14-16]. Clinicians must recognize the dual nature of fluids, utilizing bedside tools astutely to tailor fluid interventions, thus averting pathological markers of venous congestion [17]. This awareness underscores the need for a nuanced approach where clinical understanding and precise tools guide fluid management strategies, ultimately enhancing patient outcomes.

The cases presented in this study underscore the significant impact of VExUS in evaluating venous congestion across the vital vasculature, enabling tailored interventions in critical adult patients with acute kidney injury. It helped distinguish between chronic venous congestion and acute cardiac dysfunction, facilitating precise adjustments in diuretic therapy to prevent indiscriminate fluid loading and diuretic resistance. VExUS's real-time monitoring capability aligns seamlessly with the concept of precision medicine, offering personalized, evidence-based interventions based on individual patient characteristics and responses [17-19]. VExUS aids in making precise fluid management decisions, ensuring that the heart and kidneys are not further compromised by fluid overload. By offering a non-invasive, real-time assessment of venous congestion, VExUS allows clinicians to tailor fluid therapy, use diuretics effectively, and implement other interventions as needed. This individualized approach improves patient outcomes by optimizing fluid status, reducing the risk of complications, and enhancing the overall management of AKI in patients with cirrhosis, COPD, and right heart failure.

The limitations of the current study were that it was a pilot study from a single center. The treatment of the patients was decided by the treating physician in consultation with the intensivist and nephrologist. Larger studies are needed to establish the cause-and-effect relationship between the risk factors in high-risk patients and to determine if VExUS is a suitable tool to diagnose and follow venous congestion in these complex patients.

To further enhance critical care practices, it is imperative to invest in education and training initiatives for healthcare providers to harness the potential of VExUS effectively. Moreover, future research should explore its applications in larger patient cohorts and assess its long-term impact on mortality and morbidity, ushering in an era of improved, tailored patient care.

## Conclusions

VExUS emerged as a valuable tool in assessing splanchnic venous congestion, guiding fluid therapy, and improving patient outcomes. The ability to stratify venous congestion severity allowed for tailored interventions leading to optimized renal perfusion and reduced right ventricular strain. Incorporating VExUS into the diagnostic algorithm for complex AKI cases with suspected right ventricular pathology holds promise for enhancing patient care and outcomes.
